# Limited threat of *Plasmodium falciparum pfhrp2* and *pfhrp3* gene deletion to the utility of HRP2-based malaria RDTs in Northern Uganda

**DOI:** 10.1186/s12936-023-04830-w

**Published:** 2024-01-02

**Authors:** Bosco B. Agaba, David Smith, Jye Travis, Cielo Pasay, Monica Nabatanzi, Emmanuel Arinaitwe, Isaac Ssewanyana, Susan Nabadda, Jane Cunningham, Moses R. Kamya, Qin Cheng

**Affiliations:** 1 Faculty of Medicine, Department of Medical Laboratory Sciences, Mbarara University of Science and Technology, Mbarara, Uganda; 2 National Malaria Control Division, Kampala, Uganda; 3 London School of Hygiene and Tropical Medicine, London, UK; 4 Infectious Diseases Research Collaboration, Kampala, Uganda; 5 QIMR Berghofer Medical Research Institute, Kelvin Grove, QLD Australia; 6 Australian Defence Force Malaria and Infectious Disease Institute, Kelvin Grove, Australia; 7 National Health Laboratory Services/Central Public Health Laboratories, Kelvin Grove, Uganda; 8 Global Malaria Programme, World Health Organization, Geneva, Switzerland

**Keywords:** Malaria, Rapid diagnostic tests, *Plasmodium falciparum*, Histidine rich protein, *pfhrp2* and *pfhrp3* Gene deletion

## Abstract

**Background:**

Rapid diagnostic tests (RDTs) that detect *Plasmodium falciparum* histidine-rich protein-2 (PfHRP2) are exclusively deployed in Uganda, but deletion of the *pfhrp2/3* target gene threatens their usefulness as malaria diagnosis and surveillance tools.

**Methods:**

A cross-sectional survey was conducted at 40 sites across four regions of Uganda in Acholi, Lango, W. Nile and Karamoja from March 2021 to June 2023. Symptomatic malaria suspected patients were recruited and screened with both HRP2 and pan lactate dehydrogenase (pLDH) detecting RDTs. Dried blood spots (DBS) were collected from all patients and a random subset were used for genomic analysis to confirm parasite species and *pfhrp2* and *pfhrp3* gene status. *Plasmodium* species was determined using a conventional multiplex PCR while *pfhrp2* and *pfhrp3* gene deletions were determined using a real-time multiplex qPCR. Expression of the HRP2 protein antigen in a subset of samples was further assessed using a ELISA.

**Results:**

Out of 2435 symptomatic patients tested for malaria, 1504 (61.8%) were positive on pLDH RDT. Overall, qPCR confirmed single *pfhrp2* gene deletion in 1 out of 416 (0.2%) randomly selected samples that were confirmed of *P. falciparum* mono-infections.

**Conclusion:**

These findings show limited threat of *pfhrp2/3* gene deletions in the survey areas suggesting that HRP2 RDTs are still useful diagnostic tools for surveillance and diagnosis of *P. falciparum* malaria infections in symptomatic patients in this setting. Periodic genomic surveillance is warranted to monitor the frequency and trend of gene deletions and its effect on RDTs.

## Background

Malaria remains a public health problem in Uganda [1]. Transmission occurs throughout the year with peak transmission occurring between in May–June and November–December. Although the entire population remains at risk of malaria infections, transmission is heterogenous across regions [2]. Recent evidence has shown epidemiological transition such as a shift in parasitaemia from children under 5 to those aged 2–15 years as well as variations in transmission and parasite prevalence at sub-national levels [2, 3]. Although *Plasmodium falciparum* is still the predominant species and accounts for over 90% of malaria infections, *Plasmodium malariae*, *Plasmodium ovale* and *Plasmodium vivax* are also present [2]. Malaria case management that involves test and treat is a key intervention for identification and clearance of parasites in infected cases [4]. Although microscopy examination of blood smears is the gold standard diagnostic method for malaria, the use of HRP2 RDTs accounts for up to 90% of total malaria testing in Uganda [3].

Despite the current malaria control measures, the country continues to experience rise in malaria cases with frequent and protracted epidemics [3]. New biological threats have also emerged posing new challenges to country malaria control programmes. In Uganda, there are recent reports of emerging malaria parasites that evade detection by the routinely used malaria HRP2-based RDTs due to *pfhrp2* gene deletions [5–7] and those that evade treatment due to reduced sensitivity to artemisinin-based combination therapy (ACT) mediated by genetic mutations in the parasite’s *kelch13* propeller gene [8–11]. The occurrence of parasites that fail to express the HRP2 protein antigen due to deletion of *pfhrp2/3* genes has been known to cause false negative RDT results affecting malaria case management in many settings in Africa, the Amazon and India [6, 12–19]. Once the *pfhrp2/3* genes are deleted, the parasites do not express the HRP2 protein that is the principal target for RDTs resulting in false negative results [5, 20, 21]. The implication is that infected individuals remain untreated and continue transmitting parasites as infectious reservoirs but also remain at risk of progressing to severe malaria and death. HRP2-detecting RDTs are the main method for malaria diagnosis at all health facilities in the study areas of Acholi, Lango, W. Nile and Karamoja. Previous genomic surveillance has confirmed the presence of *pfhrp2/3* gene deletions in parasite populations in multiple locations in Uganda. The World Health Organization (WHO) recommends continuous surveillance to monitor the levels of deletions in areas where they have been confirmed as well as assess if the mutant parasites have emerged in other regions [5, 7].

The northern regions of Acholi, Karamoja, W. Nile and Lango in Uganda covered by this survey have traditionally remained high transmission areas at holoendemic levels compared to other malaria endemic parts of the country [22]. The HRP2 RDTs account for > 80% of total routine malaria testing in this setting. The emergence and spread of parasites with *pfhrp2* gene deletions will pose a serious threat to the test and treat strategy and likely increase malaria transmission and burden in these areas. Therefore, there is an urgent need to assess the occurrence of parasite populations with *pfhrp2* gene deletions and extent of spread in Northern regions to inform malaria test policy. The current genomic surveillance focused on investigation of *pfhrp2/3* gene deletions in symptomatic patients as recommended by the WHO [5].

## Methods

### Study design

This was a health facility-based surveillance targeting symptomatic malaria patients seeking health care as recommended by the WHO protocol for surveillance of *pfhrp2/3* deletions.

### Study area and setting

**Fig. 1:**
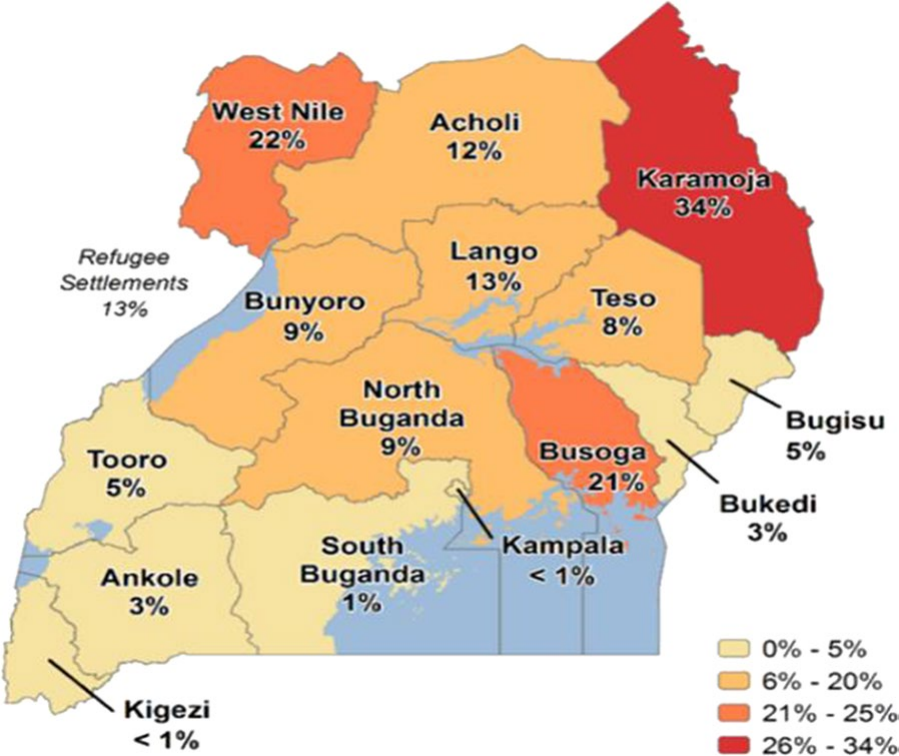
Malaria parasite prevalence by blood smear microscopy per region from population-based surveys in the study areas [22]

The survey was conducted in four regions of Acholi, Karamoja, W. Nile and Lango in northern Uganda (Fig. 1). The four regions covered by the survey are well designed demographic health survey (DHS) clusters or enumeration areas that are periodically used for the national malaria indicator surveys in the country. Malaria transmission across all the four regions is intense and stable at holoendemic levels, with parasite prevalence ranging from 13–34% as determined by blood smear microscopy (Fig. 1) [22].

### Population

The survey targeted symptomatic individuals of all age groups, suspected to have malaria who seek treatment and care at health facilities within the survey regions. Symptomatic status was based on fever defined as axillary temperature of ≥ 37.5^0^ C.

### Sampling

**Fig. 2:**
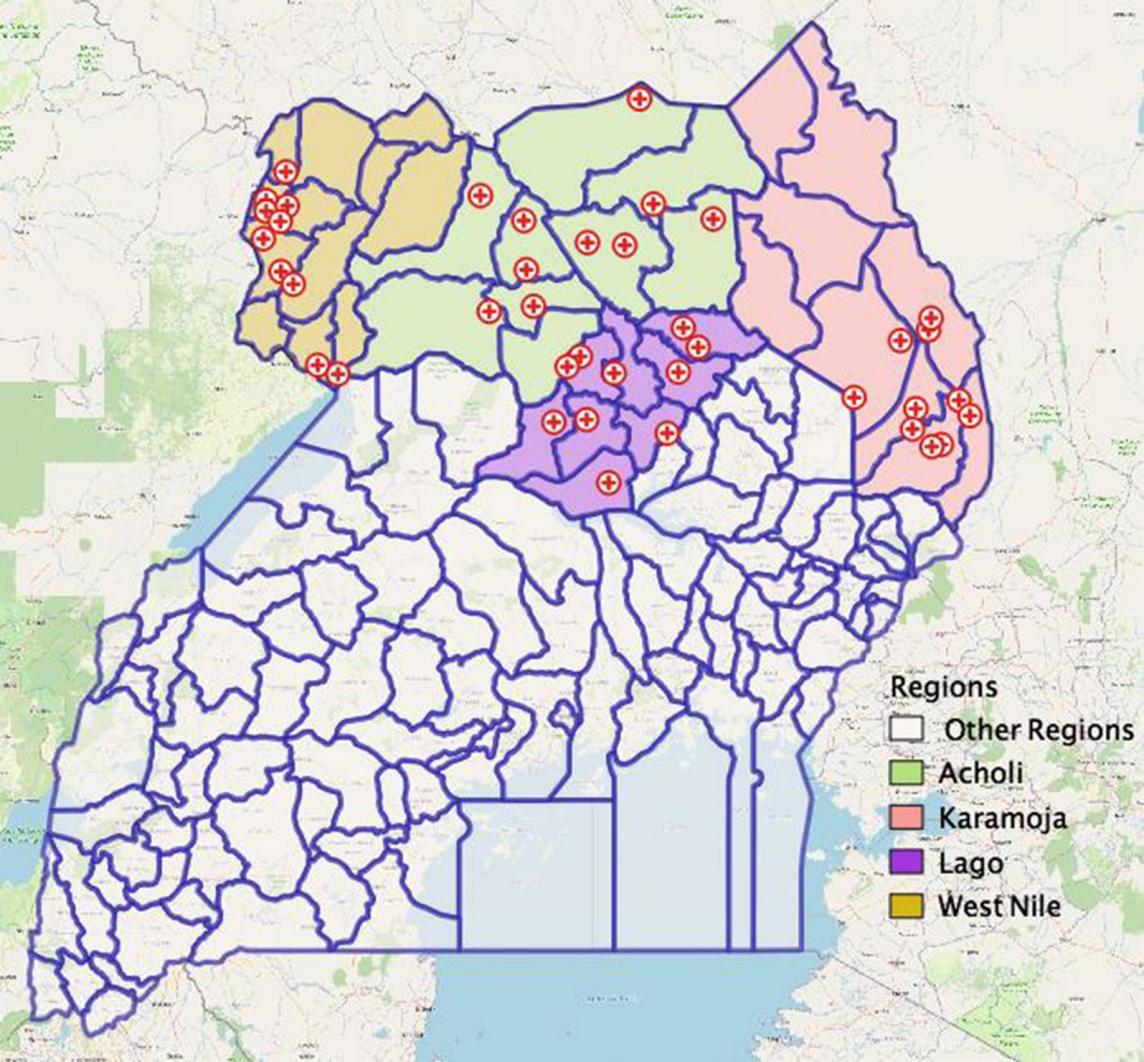
Location of survey sites

Health facilities that served as survey sites were selected in accordance with the WHO protocol for *pfhrp2/3* gene deletions surveillance [23]. Briefly, a total of ten facilities were randomly selected from each region (domain) making a total of forty facilities across the four survey regions (Fig. 2).

From each selected facility, a total of thirty-seven malaria confirmed positive blood samples from symptomatic individuals were collected on filter papers and transported to the central laboratory in Kampala for processing.

### Survey procedures and recruitment

At each facility, a designated staff explained the survey procedures and administered a consent form to eligible patients who expressed willingness to enroll. A questionnaire was used to collect patient demographics. Consenting patients were screened for malaria using two malaria rapid diagnostic tests, an HRP2 detecting and a pLDH detecting RDT. Dried blood spots (DBS) were collected from all patients with pLDH positive results.

### Eligibility criteria

All malaria suspected symptomatic individuals who presented to the selected facilities and provided consent to participate were included in the survey. Samples with negative pLDH results were excluded.

### Ethical consideration

The survey protocol was approved by the National council of science and technology (UNCST) and the Makerere University School of Public Health research and ethics committee. A consent form translated into the four indigenous languages spoken in the four different survey regions was administered for all patients before recruitment into the survey.

### Rapid diagnostic tests (RDTs)

At the health facility, two different RDTs were used and a dried blood spot collected from each patient recruited in the survey. The RDTs used were SD Bioline malaria Ag P.f Cat. 05FK50 (Abbott Diagnostics Korea Inc.) that has the Pf (HRP2) line only and the SD Bioline Malaria Ag Pf (HRP2/pLDH) Cat. 05FK90 – (Abbott Diagnostics Korea Inc.) that has a separate HRP2 and a pf-LDH test line. These tests were done simultaneously to test for malaria in symptomatic febrile patients (based on axillary temperature of > 37.5 ºC). Both RDT were used following the manufacturer’s instructions. Dried blood spot was collected from each patient recruited in the survey.

### Parasite DNA extraction

The dried blood spots were shipped to the collaborating institution, the Australia Defense Forces Malaria and Infectious Diseases Institute (ADFMIDI), a WHO Collaborating Centre for Malaria and a member of the WHO laboratory network for molecular testing to detect *pfhrp2/3* deletions, for parasite genomic analysis. From each DBS sample, 3 discs of dried blood spot were punched into sterile tubes. DNA was extracted using QIAamp DNA Mini Kits and a QIAcube Connect (QIAGEN, Crawley, UK) according to the manufacturer’s instructions. Samples were eluted into a volume of 100 µL with nuclease free water.

### Characterization of *pfhrp2* and *pfhrp3* deletions in samples

A published multiplex qPCR method that amplifies a fragment each of *pfhrp2, pfhrp3, pfldh* and human tubulin (*htb*) genes simultaneously [24] was used to determine *pfhrp2* and *pfhrp3* status in collected patient samples. Published primers, probes and cycling conditions were used with slight modifications in the probe fluorescence dye and quencher combinations including changing quencher on the *pfhrp2* probe to BHQ1 and *pfldh* probe to BHQ2. The multiplex qPCR assay uses a Quantinova Multiplex PCR kit master mix (QIAGEN) and was carried out on a Mic qPCR cycler (Bio Molecular Systems). The assay includes two internal controls: *pfldh* for the quality of parasite DNA and human tubulin gene for the efficiency of DNA extraction process. Serial diluted DNA (1, 0.1, 0.01 and 0.001 ng/µL) from a laboratory strain 3D7 (no gene deletions) was used in each run to establish standard curve for quantitation. Laboratory lines with gene deletions such as Dd2 (*pfhrp2*-deleted), HB3 (*pfhrp3*-deleted) and 3BD5 (double *pfhrp2/3* deleted) were also included in each PCR run as controls.

Cq*htb* values were used as DNA extraction control from DBS samples with a Cq*htb* value > 30 considered invalid due to inefficient extraction. Samples with no detectable *pfldh*, or Cq*pfldh* > 35 were also classified as invalid due to insufficient quality of parasite DNA (either due to low parasite counts or no parasites). Samples with ΔCq (Cq*pfhrp2*—Cq*pfldh* and Cq*pfhrp3*—Cq*pfldh*) values ≥ 3, or not detected at all are classified as *pfhrp2* and *pfhrp3* deleted [24].

### Serological analysis to confirm double *pfhrp2/3* deletions

HRPs and pLDH antigen levels were measured for samples classified as single and double *pfhrp2/3* deletions using antigen specific ELISAs (Quantimal Celisa Pf HRP2 Assay kit, KM 810 and Quantimal Celisa Pf pLDH Assay kit, KM7, Cellabs, Australia) to confirm non-expressions of HRPs while expressing pLDH. A subset of samples without gene deletions were also measured by ELISA as controls.

### Molecular species diagnosis

A conventional multiplex PCR targeting species specific 18S rRNA gene [25] was performed to confirm *Plasmodium* spp (*P. falciparum*, *P. vivax*, *P. malariae*, *P. ovale)* for all samples*.*

### Data analysis

Using a data tool, patient demographics and variables were collected from the symptomatic patients at survey sites. The data tool was a hardcopy questionnaire administered and completed by the study staff with a carbon copy to ensure its backup. All the completed hardcopy data tools were stored in lockable cupboards and later transported to Kampala for entry. All data were entered and managed in one central Excel database. Data quality checks were done to check for and correct any inconsistencies. Data analysis was done with STATA Ver 14, College Station, TX, USA: StataCorp LP. Descriptive analysis was done to describe the participant baseline characteristics and determine proportions of parasitemia and gene deletions in the samples. ArcGIS software version 10.8, Environmental Systems Research Institute (ESRI), CA, USA was used to map the locations where all blood samples were collected across the survey regions.

## Results

### Participants and RDT results

A total of 2435 symptomatic patients were enrolled and screened across 40 surveillance sites in the four survey regions using the HRP2 and pLDH RDT. Out of the 2435 malaria symptomatic patients tested, 1504 (61.8%) were positive for malaria based on the pLDH RDTs, while 1657 (68.0%) were positive on HRP2 RDT (Table 1).

**Table 1:** Demography of participants and RDT results (n = 2435)

Variables	Participants (N)	Proportion Positive (pLDH) N (%)	Proportion positive (HRP2) N (%)	HRP2-/pLDH + N (%)	HRP2 + /pLDH-N (%)	HRP2 + /pLDH + N (%)
Age						
< 5	664	437 (65.8%)	464 (69.9%)	16 (2.4%)	49 (7.4%)	420 (63.2%)
> = 5	1771	1068 (60.4%)	1193 (67.4%)	34 (1.9%)	164 (9.3%)	1034 (58.4%)
Sex						
Male	856	287 (33.8%)	234 (27.6%)	17 (2.0%)	70 (8.3%)	544 (64.2%)
Female	1579	641 (40.6%)	544 (34.4%)	45 (2.8%)	143 (9.1%)	900 (57.0%)
Region						
Acholi	549	378 (68.0%)	372 (67.8%)	7 (1.8%)	3 (0.55%)	371 (67.6%)
Lango	583	370 (63.8%)	448 (76.8%)	19 (5.1%)	99 (17.0%)	351 (60.2%)
W. Nile	676	386 (57.1%)	471 (69.7%)	13 (3.4%)	102 (15.1%)	369 (54.6%)
Karamoja	627	370 (59.0%)	366 (58.4%)	11 (3.0%)	9 (1.4%)	363 (57.8%)
Total	2435	1504 (61.8%)	1657 (68.0%)	50 (3.3%)	213 (8.7%)	1454 (59.6%)

**Fig. 3:**
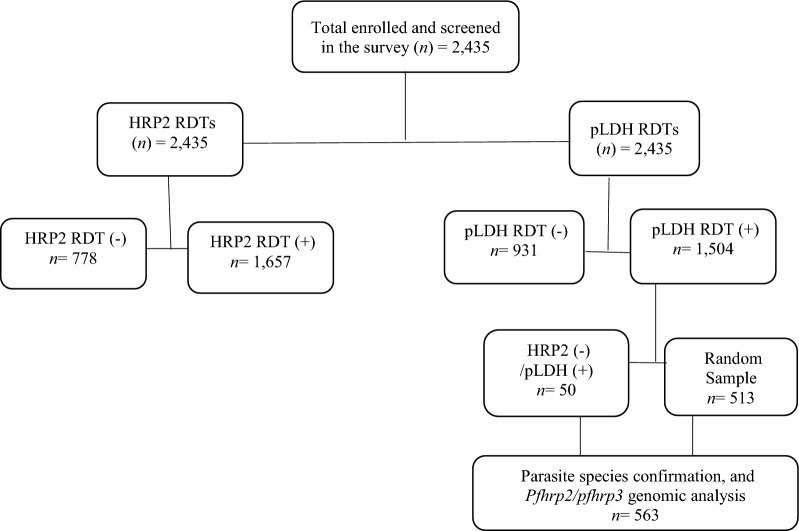
Survey Flow chat

All samples that were pLDH RDT positive were assumed to contain parasites. Of the 1504 positive pLDH RDT samples, 50 were HRP2 (-)/pLDH ( +) discordant samples giving an overall discordance (HRP2 -/pLDH +) rate of 3.3% (50/1504) in the survey (Fig. 3).

A random sample of 563 DBS were selected from the 1504 for DNA confirmation of the presence of *Plasmodium* species and subsequent investigation and characterization of the *pfhrp2/3* gene deletions (these included the 50 HRP2-/pLDH + samples). The selected samples represent 23.6% to 27.5% of the total pLDH positive samples from each region. The baseline characteristics of all survey samples (overall and per region) is shown in Tables 1, 2.

**Table 2:** Number and percentage of samples with valid qPCR results (N = 563)

Location	Total sample	Samples with valid PCR results	
	n	N	%
Acholi	140	93	66.4%
West Nile	133	113	85.0%
Karamoja	135	93	68.9%
Lango	155	117	75.5%
**Total**	**563**	**416**	**73.9%**

### Real-time multiplex qPCR

Out of the 563 samples run on multiplex qPCR, 73.9% (416/563) gave valid PCR results (Cqhtpb < 30 and/or Cqpfldh < 35) indicating sufficient human DNA and parasite DNA (Table 3). The 147/563 samples (26.1%) giving invalid PCR results (Cqhtb > 30 and/or Cqpfldh > 35) did not have sufficient human and parasite DNA to accurately determine *pfhrp2* and *pfhrp3* status and were excluded from gene deletion analysis. Invalid qPCRs were related to poor DBS resulting in poor DNA extraction (Cqhtb > 30) for 22 (3.9%) samples while the 125 samples (22.2%) was likely due to low parasite density or no parasites (Cqpfldh > 35).

**Table 3:** Number and prevalence of gene deletions in samples with valid PCR results

Location	Valid PCR	Samples without deletion		ΔCq_*pfhrp2-pfldh*_ > 3 or not detected (gene deleted parasites > 90% in the sample)	
		n	%	n	%
Acholi	93	93	100	0	0
West Nile	113	113	100	0	0
Karamoja	93	93	100	0	0
Lango	117	116	99.1	1	0.9
Total	416	415	99.8	1	0.2

### *Pfhrp2 and pfhrp3* gene status

Of 416 samples that gave valid PCR results, 1 sample (0.2%, AM44) from Lango was confirmed to have single *pfhrp2* deletion (Table 4). This was confirmed also by conventional PCR. Two other samples from Lango (AL42 and AM17) may also contain *pfhrp2* deleted parasites but not dominant (< 90%) in the sample (mixed with parasites without deletions) as ΔCq_*pfhrp2-pfldh*_ values were 1.7 and 1.9, respectively.

**Table 4:** *Plasmodium* spp confirmed by multiplex PCR

Location	Total samples tested	Samples positive	*Plasmodium* species composition in the samples (*n* = 563)							
			Pf		Po		Pf + Pm		Pf + Pm + Po	
			n	%	n	%	n	%	n	%
Acholi	140	125	125	100.0	0	0.00	0	0.00	0	0.00
West Nile	133	126	124	98.40	1	0.80	1	0.80	0	0.00
Karamoja	135	127	125	98.40	0	0.00	2	1.60	0	0.00
Lango	155	145	144	99.30	0	0.00	0	0.00	1	0.70
Total	563	523	518	99.00	1	0.20	3	0.60	1	0.20

*Pf* (*Plasmodium falciparum*)*, Po* (*Plasmodium ovale*)*, Pm* (*Plasmodium malariae*)

Discordant subset: 50 samples were positive on PfLDH and negative on HRP2 band. Only 22/50 samples (44.0%) gave valid qPCR results, i.e. had sufficient human DNA and parasite DNA, of which no *pfhrp2* and/or *pfhrp3* deletions were detected. 28 samples gave invalid qPCR results due to DBS issues resulting in insufficient human and parasite DNA for analysis while 7 was due to low parasite density or negative for parasites. All 21 samples with DBS issues also gave negative ELISA for both HRP2 and pLDH. Therefore, samples with DBS issues failed to extract DNA and failed to elute proteins. 14/21 samples with DBS issues and 4/7 low parasite samples were also negative for *Plasmodium* species PCR. Only one sample in this group was determined to have mixed infections (Pf/Po/Pm). It is not clear why samples with DBS issues were enriched in the discordant subset.

### ELISA results

HRP2 and pLDH ELISAs were performed for a subset of 125 samples including the single sample determined by both qPCR and conventional PCR as *pfhrp2*-deleted and two samples may contain *pfhrp2*-deleted parasites mixed with parasites without gene deletions. 98/125 samples including three *pfhrp2*-deleted samples gave positive ELISA results for both HRP2 and pLDH. Only 27/125 samples were negative for HRP2 of which 23 were also negative for pLDH and classified by qPCR as having insufficient DNA.

### Detection of non-*P. falciparum* species

Among 563 samples, 523 samples including 416 samples that gave valid multiplex qPCR results gave positive identifications for plasmodium species by PCR. *Plasmodium falciparum* accounted for 99.0% (518/523). One *P. ovale* (0.2%), 3 *P. falciparum* + *P. malariae* (0.6%) and 1 *P. falciparum* + *P. malariae* + *P. ovale* (0.2%) was detected (Table 4). In this subset of samples that were pfLDH RDT positive, all but one were confirmed to contain *P. falciparum* infections by PCR.

## Discussion

This is the first survey in Uganda conducted following the WHO protocol: 37 symptomatic malaria patients enrolled per facility and 10 facilities in each region; a double RDT screening method was used, and DBS collected for parasite genomic analysis [23]. Based on the WHO protocol, the survey design and sample size are good for determining if prevalence of gene deletions causing false negative RDT results is a major threat to the utility of RDTs in Uganda. The molecular analyses focused on the discordant set (n = 50) and a randomly selected set of concordant samples (n = 513). This strategy is aligned with the WHO recommendations. Following this protocol, a single sample with *pfhrp2* deletion only was identified in one of 4 regions surveyed. Two other *pfhrp2*-deleted parasites were detected in mixture with wild type parasites suggesting gene deleted parasites are circulating in Lango region. These single *pfhrp2*-deleted parasites are unlikely to have a major impact on the utility of HRP2 based RDTs because of: (1) low prevalence, (2) single *pfhrp2*-deleted parasites still expressed measurable HRP proteins likely due to cross reactivity with HRP3, (3) no gene deletions were detected from the discordant set of samples. This was also supported by the markedly higher HRP2 RDTs positive rate than that of pLDH in symptomatic patients of this study. Therefore, there is no immediate need to switch away from HRP2-detecting RDTs in Uganda as HRP2-detecting RDTs are generally more sensitive and heat durable than pLDH-detecting RDTs. However, as mathematical modelling have shown that once gene deleted parasites exist, the prevalence will rise rapidly under the continued use of HRP2-based RDTs [26]. Therefore, while no need to change RDTs at the moment, continued genomic surveillance across the country is required.

The prevalence of 0.2% *pfhrp2* deletion observed in this study among a subset of symptomatic individuals is relatively low implying limited threat to the utility of HRP2 RDTs in this setting. The assumption was that this prevalence also apply to the entire sample set of the study, as the subset of samples undergone genomic analysis were randomly selected representation 24–29% of samples collected from each region. This prevalence is relatively lower than what was previously detected in Eastern and Western regions Uganda [5]. This difference in prevalence of *pfhrp2/3* deletions between regions may be explained by the differences in the volumes of RDTs and the duration within which the RDTs have been in use since introduction. Historically, initial pilot and feasibility studies of malaria RDT use were conducted in the mid-western and eastern Uganda followed by the actual RDTs introduction, deployment and scale up to other regions in a phased manner. The emergence of *pfhrp2/3* gene deletions may occur first in areas with long term use of RDTs as this mutation allows the parasite to evade detection and survive and contribute to transmission. Regionally, the prevalence of *pfhrp2*-deleted parasites seen in the survey is also lower when compared to those reported in other endemic countries in Africa such as Eritrea [13], Ethiopia [16] and Ghana [12].

The WHO *pfhrp2/3* surveillance protocol recommends a switch of malaria RDTs from HRP2 to those targeting alternatives antigens, such as LDH when prevalence of gene deletions causing false negative RDT results exceeds the 5% cut off [23, 27]. The low prevalence of single gene deletions observed in the survey implies that the HRP2 RDTs tests are likely to detect the majority of *P. falciparum* malaria infections in symptomatic patients in this setting. However, continuous *pfhrp2/3* surveillance is recommended to monitor the trends and extent of *pfhrp2/3* deletions.

Historically, genomic characterization of *pfhrp2/3* deletions was done by conventional PCR that amplifies the exon 1 and exon 2 of the two genes and detected by gel electrophoresis. In recent years, several new molecular based approaches have been developed and adopted for the detection and characterization of *pfhrp2/3* gene deletion providing more streamlined and robust analysis and yielding more accurate results. In this survey, a published multiplex qPCR method was used that amplifies a fragment each of *pfhrp2, pfhrp3, pfldh* and human tubulin (*htb*) genes simultaneously [24]. The sample identified to have *pfhrp2* deletion was also confirmed by the conventional PCR. The WHO *pfhrp2/3* protocol also recommends proof of failure to express HRP2 protein antigen in isolates classified as *pfhrp2/3* gene deleted. However, it is well established that single *pfhrp2*- deleted parasites often test positive for HRP2 protein due to cross reactivity with HRP3. Indeed, the single sample determined by qPCR as *pfhrp2*-deleted was positive on HRP2 ELISA suggesting the presence of HRP3 protein. As there was only one sample confirmed of having single *pfhrp2*-deleted parasite in the entire sample set, there was no value to perform further ELISA on this set of samples.

## Limitations

Out of the entire survey population, only a random sample were analysed by molecular and serological methods to detect *pfhrp2/3* gene deletions and HRP2 protein expression respectively. However, significant impact on prevalence is not expected as a good proportion of samples was analysed per region (130 ~ 150 samples per region). A proportion of the HRP2-/pLDH + discordant samples gave invalid qPCR results mainly due to DBS issues resulting in insufficient human and parasite DNA for analysis. While most of the other samples eluted well and gave good quality DNA, nearly half of the DBS in the discordant set failed to lyse despite extending incubation in lysis buffer at 85° C from 15 to 30 min and 4 °C overnight. The issues encountered with the dried blood spots (DBS) particularly in the discordant set prevented molecular analysis for ~ 45% discordant samples to be analysed and this could have led to potential risk of underestimation of *pfhrp2* gene deletion in this discordant sample set.

## Conclusion and implications for the national malaria control programme

This study provides the first evidence of *pfhrp2* deletion in *P. falciparum* parasite populations circulating in Northern Uganda and the first survey to be conducted in accordance with the WHO surveillance protocol for *pfhrp2/3* deletions in Uganda. Overall, these findings show limited presence of *pfhrp2/3* gene deletions causing false negative RDT results in this setting to be below the 5% WHO recommended cut-off required for switch of RDTs. The low prevalence observed implies limited threat of *pfhrp2/3* gene deletions suggesting that the HRP2 RDTs are still useful diagnostic tools to support malaria surveillance and case management in symptomatic patients in this setting. However, confirmed presence of single *pfhrp2* gene deletion in this parasite population underscores the need to conduct periodic genomic surveillance to monitor the frequency and trend of *pfhrp2/3* gene deletions and its effect in this setting.

## Data Availability

Data for this *pfhrp2* and *pfhrp3* study is available upon request to the corresponding author. All data files related to this work have been uploaded as additional files to the manuscript.

## Abbreviations

*HRP2*: Histidine rich protein 2
*pfhrp2*: *Plasmodium falciparum* Histidine rich protein 2 gene
*pfhrp*3: *Plasmodium falciparum* Histidine rich protein 3 gene
*RDT:*: Rapid diagnostic test
*ACT:*: Artemisinin-based combination therapy
*PCR:*: Polymerase chain reaction
*WHO:*: World Health Organization
*DNA:*: Deoxyribonucleic acid
*DBS:*: Dried blood spots
*WBC:*: While blood cells
*NAAT:*: Nucleic acid amplification tests
*pLDH*: Parasite lactate dehydrogenase
*qPCR*: Quantitative Polymerase Chain Reaction
*ELISA:*: Enzyme Linked Immunosorbent Assay
*DHS:*: Demographic Health Program
